# A Method for Evaluating Hunger and Thirst in Monkeys by Measuring Blood Ghrelin and Osmolality Levels

**DOI:** 10.1523/ENEURO.0481-23.2024

**Published:** 2024-08-21

**Authors:** Yuki Suwa, Jun Kunimatsu, Akua Kamata, Masayuki Matsumoto, Hiroshi Yamada

**Affiliations:** ^1^Academic Service Office for the Medical Science Area, University of Tsukuba, Tsukuba 305-8577, Japan; ^2^Division of Biomedical Science, Institute of Medicine, University of Tsukuba, Tsukuba 305-8577, Japan; ^3^Transborder Medical Research Center, University of Tsukuba, Tsukuba 305-8577, Japan; ^4^Medical Sciences, University of Tsukuba, Tsukuba 305-8577, Japan

**Keywords:** hunger, monkey, reward, satiety, thirst

## Abstract

Hunger and thirst drive animals’ consumption behavior and regulate their decision-making concerning rewards. We previously assessed the thirst states of monkeys by measuring blood osmolality under controlled water access and examined how these thirst states influenced their risk-taking behavior in decisions involving fluid rewards. However, hunger assessment in monkeys remains poorly performed. Moreover, the lack of precise measures for hunger states leads to another issue regarding how hunger and thirst states interact with each other in each individual. Thus, when controlling food access to motivate performance, it remains unclear how these two physiological needs are satisfied in captive monkeys. Here, we measured blood ghrelin and osmolality levels to respectively assess hunger and thirst in four captive macaques. Using an enzyme-linked immunosorbent assay, we identified that the levels of blood ghrelin, a widely measured hunger-related peptide hormone in humans, were high after 20 h of no food access (with *ad libitum* water). This reflects a typical controlled food access condition. One hour after consuming a regular dry meal, the blood ghrelin levels in three out of four monkeys decreased to within their baseline range. Additionally, blood osmolality measured from the same blood sample, the standard hematological index of hydration status, increased after consuming the regular dry meal with no water access. Thus, ghrelin and osmolality may reflect the physiological states of individual monkeys regarding hunger and thirst, suggesting that these indices can be used as tools for monitoring hunger and thirst levels that mediate an animal's decision to consume rewards.

## Significance Statement

Standard methods for behavioral and neurophysiological experiments in nonhuman primates rely on controlled access to food or fluid rewards to motivate their performance. We previously assessed the thirst state of monkeys by measuring blood osmolality, the most widely used hematological index of hydration status. Here, we assessed the hunger state of monkeys by measuring blood ghrelin levels, a widely measured hunger-related peptide hormone in humans, using an enzyme-linked immunosorbent assay. We measured these indices and found that they reflected the hunger and thirst states of the monkeys before and after consuming dry meals, with no relation to each other. Thus, these two physical indices can be utilized to monitor hunger and thirst in primates.

## Introduction

Hunger and thirst are fundamental constituents of the physiological needs that drive an animal's consumption behavior to maintain its physical state (Mattes, 2010). Controlled access to food or drink is commonly utilized in standard experimental procedures to motivate performance in nonhuman primates. Hunger drives the consumption of food that maintains the metabolic state, while thirst drives the intake of fluid that maintains the hydration state (Berne and Levy, 1993). However, these physiological needs are not independent of each other (e.g., food items contain both energy and fluid; Ramachandran and Pearce, 1987; Watts, 1999; Betley et al., 2015). For instance, thirsty animals are unlikely to eat dry food, even if they are hungry (Eiselt et al., 2021), which implies that the reward values of food and beverages may be linked to the reward circuitry (Haber and Knutson, 2010). Hunger and thirst levels are subjectively evaluated and controlled by experimenters, yet these states vary individually due to differences in homeostatic conditions. As such, establishing a method to reliably evaluate the hunger and thirst states of animals is useful, as it drives their consumption behavior to appropriately maintain their physical state.

In the experimental testing of behavior in monkeys and rodents, typical procedures generally involve the control of either of these physiological needs to motivate the animal's performance, especially in experiments involving electrophysiology in monkeys (Evarts, 1968; Wurtz, 1968; Watanabe, 1996). To assess thirst, the blood osmolality level can be used as the standard hematological index to detect hydration status in mammals (B. J. Rolls and Rolls, 1982; Stedman, 2006). We previously used the osmolality level to relate the behavioral characteristics of economic decisions such as risk preferences (Yamada et al., 2013), instrumental performance of actions to earn fluid rewards (Minamimoto et al., 2012), and simple eye movements for fluid reward intake (Yamada et al., 2010) in monkeys, which are close relatives of humans. To assess hunger, various physical measures can be employed (Mattes, 2010), such as blood sugar levels and insulin as standard measures and leptin and ghrelin as recently developed measures. These physical indices of hunger and thirst are useful for assessing and controlling the motivational state of primates, in addition to other possible measures, such as accumulated earned rewards (Minamimoto et al., 2012; Pastor-Bernier et al., 2021). However, the lack of a simultaneous assessment of hydration and metabolic status leads to difficulty in evaluating neural correlates for hunger and thirst in behaving monkeys, as control of either hunger or thirst can affect the status of the other (Ramachandran and Pearce, 1987; Watts, 1999; Betley et al., 2015).

This aforementioned limitation introduces several potential problems in neuroeconomic studies (Camerer et al., 2005; Glimcher et al., 2008; Yamada et al., 2021; Imaizumi et al., 2022; Tymula et al., 2023) that employ experimental testing of reward valuation systems for economic choices. When measuring neural activity in the reward circuitry, the subjective values of any reward depend on the physical state of the subject (Nakano et al., 1984; Critchley and Rolls, 1996; de Araujo et al., 2006; Pritchard et al., 2008), even for money (Symmonds et al., 2010). Hunger and thirst may affect neural activity in specific brain regions (Yaxley et al., 1985; E. T. Rolls et al., 1988) or dramatically change the activity in many parts of the brain (de Araujo et al., 2006). Therefore, the control and monitoring of these states are insufficient for precisely assessing the neural valuation system embedded in the reward circuitry. The subjective values of the items used in the monkey experiments depend on these physiological statuses. For instance, the intake of juice rewards must elicit changes in both statuses since it contains both sugar and liquid. Thus, assessing hunger and thirst with controlled access to either food or beverages is worthwhile.

In the present study, we measured blood ghrelin levels to assess hunger (Kojima et al., 1999) and osmolality to assess thirst (B. J. Rolls and Rolls, 1982) in captive monkeys. We evaluated the physical status of the monkeys using a typical experimental procedure to control food and fluid access with assessments of food and water intake behavior. Our results indicate that these indices can be used to monitor hunger and thirst in individual monkeys.

## Materials and Methods

### Experimental subjects

Five macaque monkeys were used in this study (*Macaca fuscata*; Monkey MON, male, 7.8 kg, 4 years; Monkey Y1, male, 6.4 kg, 2 years; Monkey MIY, female, 5.8 kg, 4 years; Monkey Y23, male, 6.0 kg, 4 years; *Macaca mulatta*; Monkey SUN, male, 7.5 kg, 13 years). Blood samples were collected from all four monkeys, excluding monkey Y23. The Animal Care and Use Committee of the University of Tsukuba approved all the experimental procedures (protocol number H30.336), which were also performed in compliance with the United States Public Health Service's Guide for the Care and Use of Laboratory Animals.

### Blood collection

The monkeys were anesthetized using medetomidine (0.03 mg/kg, i.m.) and midazolam (0.3 mg/kg, i.m.), except Monkey SUN who was seated in a standard primate chair and desensitized to leg restraint through positive reinforcement with food rewards before starting the blood collection procedure. Blood samples (2.0 ml) were drawn from the saphenous vein using a butterfly needle (22 Gauge) in a single collection. Atipamezole (0.024 mg/kg, i.m.) was administered, and 30 min after recovery, the monkeys were fed a dry meal at ∼10:00 until 11:30 (1.5 h), after which any remaining food was extracted. After 1 h (a total of 3 h from the first blood collection), a second blood sample was collected using the same procedure as the first sample collection. Monkey SUN was sampled while awake, exhibiting no distress during the sampling procedure after desensitization. Approximately 1.0 ml plasma was extracted in each collection from the 2.0 ml blood sample in ethylene diamine tetra-acetic acid 2K-containing tubes by centrifugation at 2,000 × *g* for 10 min at 4°C. The total amount of blood extracted within any 2 week experimental period did not exceed 5% of the total blood volume (total blood volume was estimated at 65 ml/kg weight).

Sixteen blood collections were performed over 8 d for each monkey (two blood collections per day, before and after food intake). A total of 64 blood samples were therefore collected from four monkeys.

### Regular feeding

In the approved controlled food access protocol, the monkeys received a fixed daily allocation of dry meals (certified diet, PS-A, Oriental Yeast), containing water (8.0 ± 0.7 g), protein (21.5 ± 0.3 g), fat (6.8 ± 0.3 g), ash (7.7 ± 0.2), fabric (3.1 ± 0.3), soluble nitrogen-free (52.9 ± 0.7), and calorie (359.1 ± 3.5 kcal) per 100 g. The diet also contains sodium (0.66 g per 100 g), vitamins, and other minerals. This diet was provided at least once a day at approximately noon, except during the periods before and after surgery. From 10:00 to 11:00, food and water were restricted, although before 10:00 some monkeys had finished eating food and drinking beverages on the day after the last feeding. The fixed daily allocation of food was determined by veterinary stuffs as follows: Monkey MON, 140–160 g; Monkey Y1, 140 g; Monkey MIY, 130–160 g; and Monkey SUN, 140 g; and Monkey Y23, 130 g. The average amount of dry meal consumed in a day was as follows: Monkey MON, 140 g; Monkey Y1, 136 g; Monkey MIY, 153 g; Monkey SUN, 134 g; and Monkey Y23, 129 g.

### Controlled access to food and water

**Figure 1.**
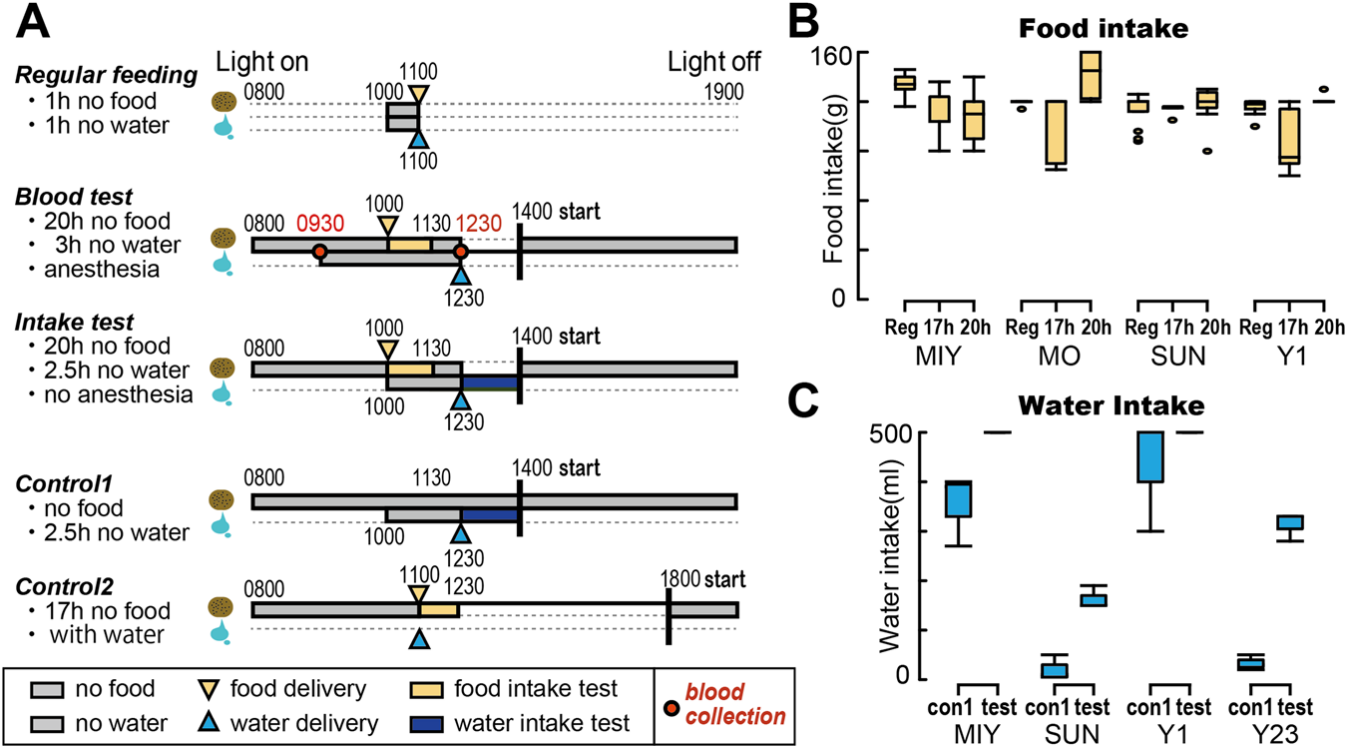
Controlled food and water intake and behavioral measures. ***A***, Five different experimental schedules for the control of food and water intakes: top row, regular feeding condition for daily allocation of the regular dry meal and water (Reg in ***B***); second row, blood test condition for collecting blood for testing the influence of hunger and thirst (20 h in ***B***); middle row, intake test condition to measure the food and water intake without anesthesia (test in ***C***); fourth row, control condition for evaluating thirst to measure the effect of dry meal intake on water intake (con1 in ***C***); bottom row, control condition to precheck whether 17 h of no food access was enough to induce the hunger, compared with the 20 h (17 h in ***B***). The controlled food and water access is initiated on the day before the test day, at 14:00, except Control 2 condition. ***B***, The amount of food intake in four monkeys during the blood test condition (20 h), Control 2 (17 h), and regular feeding with a constant dry meal applied at 11:00 (Reg). ***C***, Amount of water intake in four monkeys during the intake test (test) and control Condition 1 (con1). In ***B***, the result from monkey Y23 was not demonstrated because his blood data were not collected.

Under the experimental conditions employed (Fig. 1*A*, blood test), all leftover food was removed at 14:00 on the day before blood collection, resulting in ∼20 h of no food access. Food was delivered after the first blood collection in each individual, and the amount was allocated daily to each monkey at ∼10:00 A.M. The average amount of food consumed by each monkey during the test period was as follows: Monkey MON, 159 g or 20 g/kg/day; Monkey Y1, 140 g or 22 g/kg/day; Monkey MIY, 127 g or 22 g/kg/day; and Monkey SUN, 134 g or 18 g/kg/day. If the monkeys did not start eating the dry meal quickly, they were fed small pieces of sweet potatoes (<10 g). The monkeys had an *ad libitum* access to a 500 ml bottle of water in a day, and no water access control was performed throughout the entire period of the test that the monkeys engaged in, except for the period between the first and second blood collections. This procedure mimicked a typical experimental procedure for controlling food access, which motivated subjects to obtain food rewards, but not for liquid rewards in the experimental room.

### Test schedules for the control of hunger and thirst levels

We prepared the blood test schedule as described above with anesthesia (Fig. 1*A*, blood test) and further conducted a food/water intake test without anesthesia at the same feeding interval, but without performing blood collection (Fig. 1*A*, intake test). In these tests, controlled access to food and water was evaluated by using control conditions (Fig. 1*A*, Controls 1 and 2). Twenty hours of no food and *ad libitum* access to water was followed by food access without water for blood collection (Fig. 1*A*, second row). The same feeding interval was used in the food and water intake tests without anesthesia (Fig. 1*A*, third row). Under control Condition 1 (Fig. 1*A*, fourth row, Control 1), monkeys were not provided food to measure water intake, which confirmed that the monkeys became thirstier after consuming a dry meal. In control Condition 2 (Fig. 1*A*, bottom row, Control 2), a short feeding delay (17 h) was used to precheck the amount of food intake while drinking water when eating dry meals, which confirmed whether blood and food intake test conditions yield hunger in monkeys.

### Body weight measurements

The body weight of each monkey was measured before the first blood collection.

### Measuring the blood ghrelin levels using enzyme-linked immunosorbent assay (ELISA)

Unacylated ghrelin levels were measured using ELISA (Unacylated Ghrelin (human) Express Enzyme Immunoassay kit, Bertin Pharma, A05119.96 wells). All immunoassays were performed according to the manufacturer's instructions. The plates coated with the primary antibody in each well were rinsed five times with the wash buffer (300 µl/well) included in the kit. Standards, controls, and samples were added to each well. Additionally, 100 µl of a tracer was dispensed to the wells, and the plates were incubated at room temperature (RT) for 3 h (∼20–25°C). After rewashing, 200 µl of the detection reagent was dispensed into the wells. The plates were subsequently incubated in the dark at RT for 1 h, and absorbance was measured at 414 nm using a microplate reader (Varioskan LUX multimode microplate reader, Thermo Fischer Scientific). The ghrelin concentration in each plate was estimated from a standard curve. We used a linear fit because almost no curvilinear fit exists in standard measures.

### Measuring blood osmolality

Blood osmolality was measured for each sample; 250 µl plasma was extracted from each sample, and the osmolality was measured using a freezing point method (Advance 3250, Advanced Instruments), as described in our previous studies (Yamada et al., 2010, 2013; Minamimoto et al., 2012). The measurement error, evaluated as the value range for the same blood sample, was almost 2 mOsm/kgH_2_O. This error was determined by measuring three samples from a single collected sample, with 2 mOsm/kgH_2_O being the maximum difference observed in our previous study (Yamada et al., 2010).

### Statistical analysis

Changes in food and water intake were analyzed using a two-way analysis of variance (ANOVA) at *p* < 0.05, and the amount of dry meal consumed (*Y*) was analyzed using the following equation:

$$Y=b_{1}\mathrm{Duration}+b_{2}\mathrm{Monkey}+b_{3}\mathrm{Interaction}+\mathrm{error},$$

where errors are the residuals, “Duration” is a categorical variable used to define the duration without foods, “Monkey” is a categorical variable identifying the four monkeys, and “Interaction” is the interaction between “Duration” and “Monkey.” If *b*_1_ or *b*_3_ was not 0 at *p* < 0.05, we concluded that food duration significantly affected monkey's food intake.

The amount of consumed water (*Y*) was analyzed using the following equation:

$$Y=b_{1}\mathrm{Condition}+b_{2}\mathrm{Monkey}+b_{3}\mathrm{Interaction}+\mathrm{error},$$

where errors are the residuals, “Condition” is a categorical variable to define the test condition of the intake test and its control (Control 1), “Monkey” is a categorical variable identifying the four monkeys, and “Interaction” is the interaction between “Condition” and “Monkey.” If *b*_1_ or *b*_3_ was not 0 at *p* < 0.05, we concluded that the test conditions (i.e., food intake before the water intake test) significantly affected the water intake of the monkey.

Changes in plasma ghrelin levels before and after eating the dry meal were analyzed using two-way ANOVA at *p *< 0.05, and plasma ghrelin (*Y*) was fitted using the following equation:

$$Y=b_{1}\mathrm{Hunger}+b_{2}\mathrm{Monkey}+b_{3}\mathrm{Interaction}+\mathrm{error},$$

where errors are the residuals; “Hunger” is a categorical variable to define the hunger state, differentiated by the conditions before or after food intake on a test day; “Monkey” is a categorical variable identifying the four monkeys; and “Interaction” is the interaction between “Hunger” and “Monkey.” If *b*_1_ or *b*_3_ was not 0 at *p *< 0.05, we concluded that the hunger state significantly affected plasma ghrelin levels.

Changes in plasma osmolality levels were analyzed using two-way ANOVA at *p *< 0.05. The plasma osmolality (*Y*) was fitted using the following equation:

$$Y=b_{1}\mathrm{Hunger}+b_{2}\mathrm{Monkey}+b_{3}\mathrm{Interaction}+\mathrm{error},$$

where errors are the residuals; “Hunger” is a categorical variable to define the hunger state, differentiated by the conditions before or after food intake on a test day; “Monkey” is a categorical variable identifying the four monkeys; and “Interaction” is the interaction between “Hunger” and “Monkey.” If *b*_1_ or *b*_3_ was not zero at *p *< 0.05, we concluded that the hunger state significantly affected plasma osmolality.

### Influence of plasma concentration on the optical density measurements

The nonspecific effect of plasma on the measured optical density was evaluated by adding 5 and 10% plasma to the standard concentration. All these plasma samples were obtained from a monkey on a single day. We partially evaluated the degree of noise from the plasma sample by comparing the fitted curves to these data (5 and 10%) with a standard curve without plasma (0%). In this evaluation, we used a sample from monkey SUN, which contained relatively low ghrelin levels among the four monkeys.

The influence of the plasma concentration on the optical density was evaluated using a general linear model. The optical densities (*Y*) were fitted using the following equation:

$$Y=b_{0}+b_{1}\mathrm{COS}+b_{2}\mathrm{PC}+b_{3}\mathrm{Interaction}+\mathrm{Error},$$

where *b*_0_ and the error are the intercept and residual, respectively. “COS” is a concentration of the standard, “PC” is the plasma concentration (0, 5, or 10%), and “Interaction” is the interaction between “COS” and “PC.” If *b*_2_ was not zero at *p *< 0.05, we concluded that the plasma concentration significantly affected the optical density. *b*_2_ should be significantly different from zero if the plasma sample contains endogenous ghrelin. If *b*_3_ was not zero at *p *< 0.05, it indicated that a nonspecific effect of the plasma was present on the measured optical density in the sample.

We suspected a nonspecific effect of the plasma on the measured optical density in the following way. Assuming no endogenous ghrelin was present in the sample and that all the measured optical densities came from the plasma, the increase in the intercept values from 0 to 10% plasma indicated the amount of noise from the plasma sample. Because it is not possible to extract endogenous ghrelin from the sample, the maximum possible noise level was estimated in this way.

## Results

### Effect of controlled food and water access on consumption behavior in monkeys

To confirm that our experimental protocol obeying controlled access schedules adequately evoked hunger and thirst in monkeys, we prepared five different conditions in our experiment (Fig. 1*A*). First, in the blood test condition (Fig. 1*A*, second row), no food access was provided to the monkeys for 20 h. This controlled food access was started at 14:00 the day before the test in all test condition, except Control 2 (Fig. 1*A*, bottom row). This feeding interval of 20 h no food yielded almost the same, but the slightly different amount of dry meal's intake as that in regular feeding condition in some monkeys (Fig. 1*B*, 20 h and Reg; two-way ANOVA; *n* = 104; df = 92; duration, *F* = 13.6; *p *< 0.001; monkey, *F* = 3.10; *p *= 0.032), while the intake duration was 1.5 h for food consumption (Fig. 1*A*, second row, yellow bar). Indeed, 17 h of no food access we made as a precheck (Fig. 1*A*, bottom row, Control 2) seemed to be sufficient to elicit this level of consumption behavior (Fig. 1*B*, 17 h), although the effect of the no food duration differed between monkeys (interaction, *F* = 6.60; *p *< 0.001). Thus, our controlled food access protocol used for the blood test (20 h of no food access) adequately elicited a hunger state in the monkeys.

In the blood test, the monkeys consumed dry meals without any liquid reward (Fig. 1*A*, second row, gray bar for water). Under this feeding condition, monkeys became thirstier as indicated by the increased water intake (Fig. 1*C*, test; Fig. 1*A*, middle row) than those when they did not consume the dry meal (Fig. 1*C*, con1; Fig. 1*A*, fourth row; two-way ANOVA; *n* = 24; df = 16; condition, *F* = 58.3; *p *< 0.001; monkey, *F* = 78.9) maybe because the dry meal contained almost no water but some sodium (see Materials and Methods). Conformation of this consumption behavior without anesthesia in the intake test indicated that under the blood test condition, which employs the same schedule as the intake test condition, monkeys were hungry and thirsty at the first and second blood collections, respectively.

### Effect of dry meal intake on blood level measurements of ghrelin and osmolality

We measured the blood levels of ghrelin and osmolality before and after consuming the dry meal under the no water access conditions (Fig. 1*A*, second row) during an ∼3 h period until the second blood collection, consisting of periods of 0.5 h before start eating, 1.5 h during eating, and 1.0 h after eating the meal. This controlled food access procedure mimicked a typical experimental condition for measuring animal behavior in an experimental room that motivates subject performance to obtain food rewards, whereas the hydration status was not controlled to motivate subject performance. Two consecutive blood collections were performed on each test day with controlled food access, which was started a day before the test day and continued until the second blood collection (Fig. 1*A*, second row, blood test). Throughout the test period for each monkey, water access was not controlled, except for the period between the first and second blood collections. Plasma was extracted from whole blood samples using a standard procedure (see Materials and Methods).

**Figure 2.**
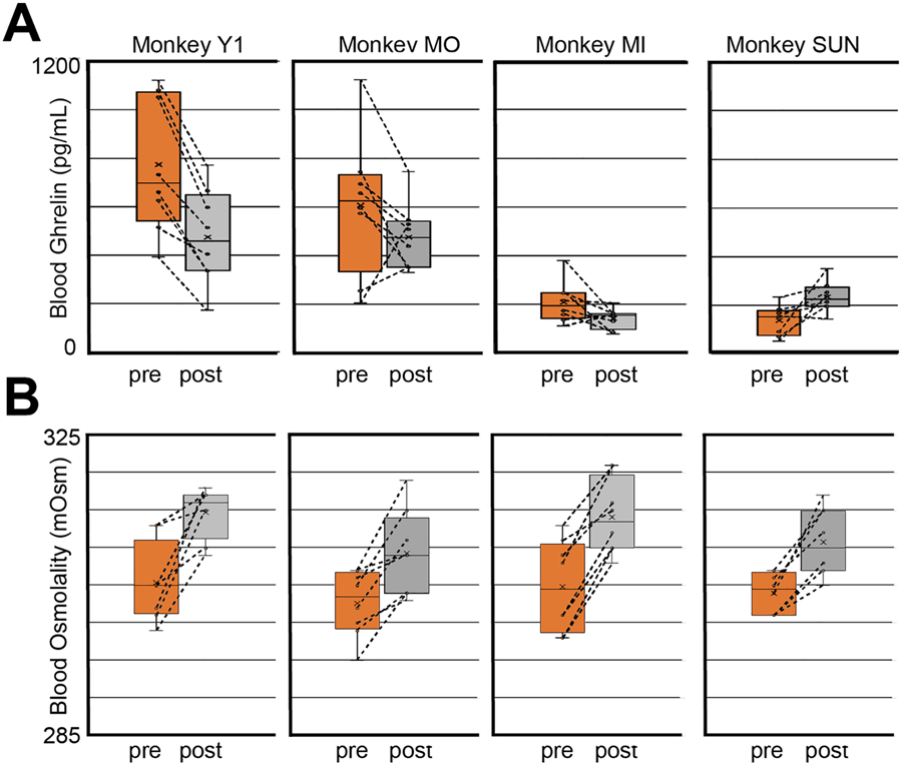
Blood ghrelin and osmolality levels before and after dry meal intake. ***A***, The box plot of blood ghrelin levels in the four monkeys before (pre) and after (post) consuming the regular dry meal. ***B***, The box plot of blood osmolality levels before (pre) and after (post) the consumption of regular dry meals in four monkeys. In ***A*** and ***B***, the mean is indicated by a cross. Consecutive measurements are obtained daily using a line.

We identified that the unacylated ghrelin levels tested using ELISA (see Materials and Methods) decreased on average after consuming the dry meal compared with those before intake (Fig. 2*A*; two-way ANOVA; *n* = 64; df = 56; hunger, *F* = 5.44; *p *= 0.023; monkey, *F* = 29.8; *p *< 0.001) in three out of the four monkeys. Additionally, blood ghrelin levels were vastly different among the monkeys (in pg/ml: Monkey SUN, 54–350; Monkey MIY, 79–380; Monkey Y1, 174–1,122; Monkey MO, 206–1,123). Monkey Y1 exhibited a decrease in value in all eight tests, while Monkeys MO and MIY exhibited a decrease in six of the eight measurements. In contrast, Monkey SUN, who exhibited the lowest blood ghrelin levels, demonstrated the opposite effect, exhibiting an increase in blood ghrelin levels (interaction, *F* = 3.61; *p *= 0.019) in seven of the eight measures. Thus, although individual differences existed, food intake consistently decreased blood ghrelin levels, except in one monkey.

To examine the effect of dry meal intake on thirst levels, we measured blood osmolality using the same blood samples described above. Our simple prediction for this result was that the monkeys would become thirstier after eating a dry meal without fluid intake, which is a typical condition when using food rewards to motivate monkeys in an experimental room. After the dry meal intake without water intake, all four monkeys exhibited consistent increases in blood osmolality (Fig. 2*B*; two-way ANOVA; *n* = 64, df = 56; Hunger, *F* = 45.7, *p *< 0.001). The effect of dry meal intake on blood osmolality differed from that on blood ghrelin levels, with no significant individual differences in osmolality levels and no significant interaction between food intake and the monkeys (monkey, *F* = 2.61; *p *= 0.060; interaction, *F* = 0.41; *p *= 0.746). Indeed, none of the samples collected over the 32 testing days (8 testing days × four monkeys) demonstrated a decrease in osmolality. Thus, the blood osmolality level was capable of consistently reflecting the internal state of thirst in all four animals (Figs. 1*C*, 2*B*), which is consistent with the results of our previous studies (Yamada et al., 2010, 2013; Minamimoto et al., 2012).

**Figure 3.**
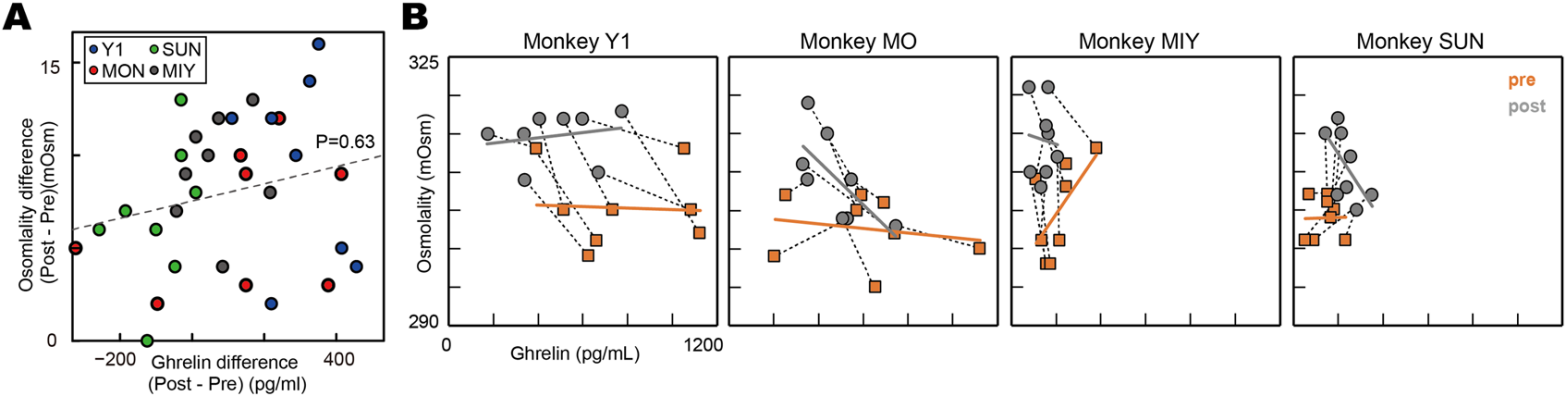
No consistent relationship between the changes in blood ghrelin and osmolality levels. ***A***, No significant relationship observed between changes in blood ghrelin and osmolality levels before (pre) and after (post) the food intake. The dashed line represents the regression slope. ***B***, Two consecutive measurements per day are represented by dotted black lines. The thick gray and orange lines indicate the regression lines for the data in the pre- and postperiods, respectively.

**Figure 4.**
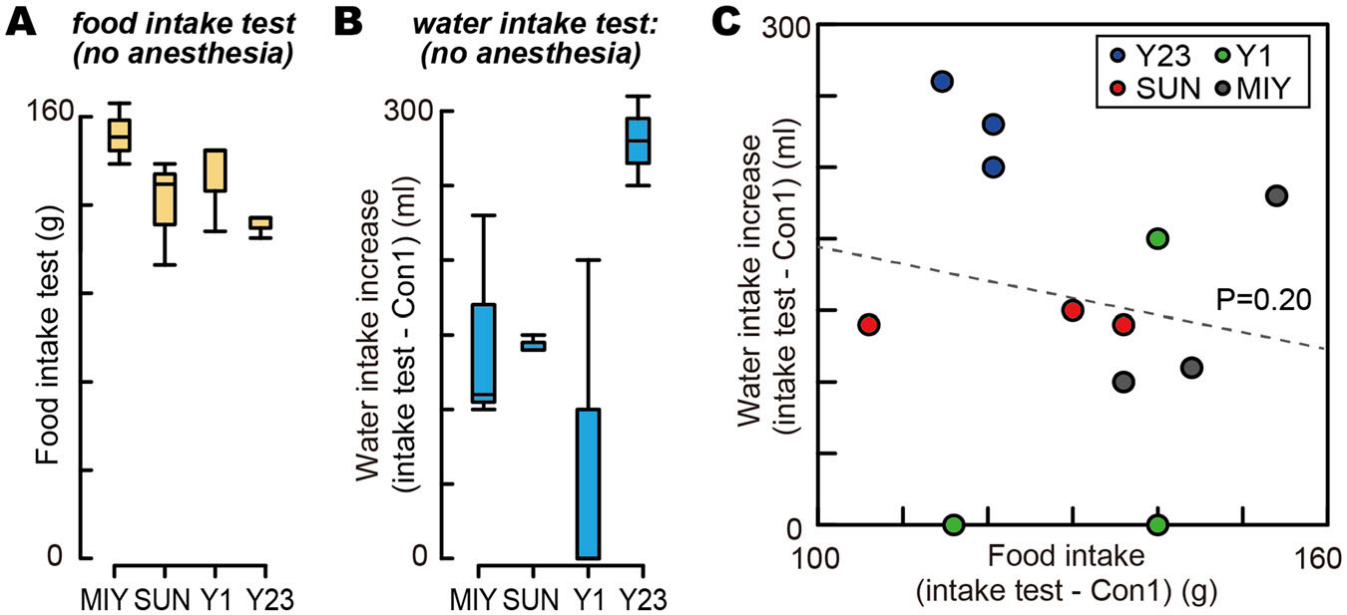
Amounts of food and water intake without anesthesia. ***A***, The amount of the dry meal intake during the 1.5 h of the food intake test. ***B***, The increase in water intake amount during the 1.5 h of the food intake test compared with that during control Condition 1, in which no food was provided. ***C***, No significant relationship observed between the increase in water intake compared with control Condition 1 (vertical axis) and the amount of the food intake during the intake test (horizontal axis). The dashed line represents the regression slope.

We further examined the relationship between changes in blood osmolality and ghrelin levels to determine whether these values were related, identifying no relation between these changes (Fig. 3*A*; linear regression, *n* = 32; df = 30; intercept, *t* = 9.54; *p *< 0.001; ghrelin difference, *t* = 1.30; *p *= 0.20). Furthermore, we visualized the relationship between the changes in osmolality and ghrelin levels in each period before and after consuming the dry meal (Fig. 3*B*) and examined whether a relationship existed between osmolality and ghrelin levels using regression analysis (Fig. 3*B*, regression slope). Overall, we noted no consistent effects among the monkeys. Monkeys Y1, MO, and MIY did not demonstrate consistent regression slopes, although these three individuals consistently exhibited decreased blood ghrelin levels and increased blood osmolality (Fig. 2*A*,*B*). Monkey SUN, which exhibited the opposite effect on blood ghrelin levels, demonstrated regression coefficients similar to those of Monkey MO. We further examined whether a behavioral relationship was present between food intake and an increase in water intake under nonanesthetized conditions (Fig. 4*A*,*B*), but did not detect any such relationship (Fig. 4*C*). Thus, blood ghrelin and osmolality levels did not covary with each other either before or after consuming dry meals, indicating that these two physical measures can be used to evaluate hunger and thirst in animals.

In summary, after dry meal intake without water, blood ghrelin levels decreased, except in Monkey SUN, whereas osmolality increased in all four monkeys. However, we did not observe a clear relationship between blood osmolality and ghrelin levels. These data indicated that the accuracy of the measurements and physical changes differed for each individual.

### Assessing the influence of plasma concentration on optical density measurement

We further evaluated the degree of measurement error (or noise) included in our ghrelin measurements from the plasma, enabling us to compare the two different physical measures of the indices for hunger and thirst, accounting for both measurement errors. For the osmolality measures we employed, we previously evaluated the measurement error of osmolality, as the value range from a single sample was within almost 2 mOsm/kgH_2_O in our procedure. Here, we assessed the degree of error in the optical measurement of unacylated ghrelin by ELISA. As plasma must induce a nonspecific effect on optical absorption, such as the nonspecific binding of proteins (Neutens et al., 2021), we evaluated the noise as follows: plasma samples were compared with different concentrations obtained from one monkey (SUN) among a single collected samples after the standard was added to them at different dilutions (0% or no endogenous plasma, 5%, and 10%). We compared these three optical measures, which included the original standard and endogenous ghrelin in the 5 and 10% plasma samples. Ideally, the fitted lines should change consistently as the percentage increases. We note that removing endogenous ghrelin from plasma is impossible, and optical absorption is derived from endogenous ghrelin, plasma, and standard ghrelin.

**Figure 5.**
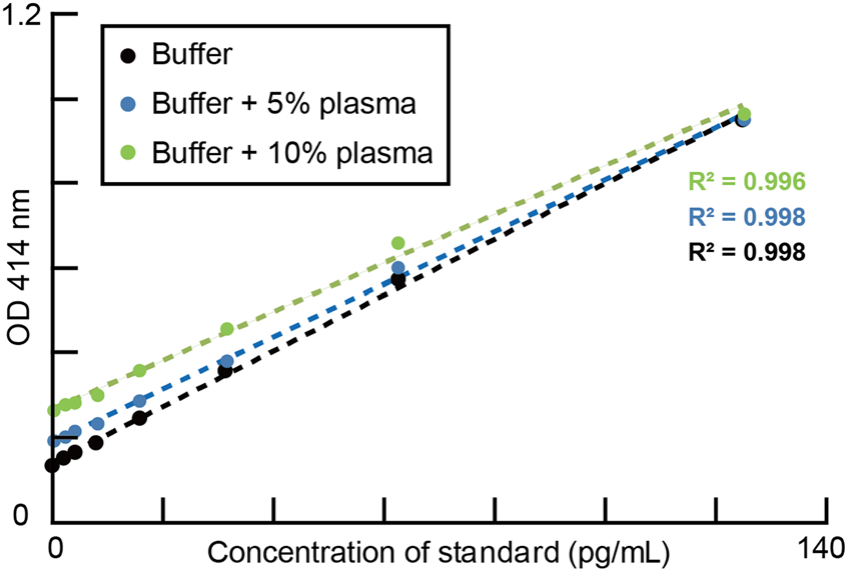
Assessing the influence of plasma concentration on measuring optical density. Plots of the measured optical density (vertical axis) against standard concentrations. Each point is obtained from the average of two wells: no plasma (black), 5% plasma (blue), and 10% plasma (green). The colored dotted lines indicate a linear fit to the data. *R*^2^ are indicated.

All three curves were well described with a linear fit because the *R*^2^ values were >0.99, indicating that 5 and 10% plasma and endogenous ghrelin elicited a consistent increase in optical absorption (Fig. 5). The background optical absorption without ghrelin or plasma was ∼0.141, as estimated from the fitted curves (intercept of the black regression slope). These values increased to 0.192 and 0.269 in 5 and 10% ghrelin-containing buffers, respectively, representing increments of 0.051 and 0.077 from 0 to 5 and 5 to 10%, respectively. This nonlinear effect was a 1.5-fold increase (0.077/0.051) and may be elicited by a 5% point increase in the plasma concentration from 5 to 10%. Moreover, the relative influence of the additive plasma on the optical measurements decreased as the amount of standard ghrelin increased (almost no differences were observed at 125 pg/ml): the optical densities were 0.949, 0.952, and 0.966 in 0, 5, and 10% plasma at 125 pg/ml, respectively. The slopes of the regression coefficients in these three linear fits became slightly shallow as the plasma concentration increased (general linear model, *n* = 24; df = 20; the concentration of standard, regression coefficient, 0.0128; *t* = 55.8; *p *< 0.001; plasma concentration, regression coefficient, 0.0066; *t* = 13.7; *p *< 0.001; interaction, regression coefficient, −0.00008; *t* = −4.6; *p *< 0.001). Thus, the blood ghrelin levels in our samples were reliably measured by ELISA using 10% plasma. In addition, some small nonspecific optical signals were derived from the plasma.

Finally, the maximum possible noise levels in the optical measurements were estimated. If we assume that no endogenous ghrelin is present in the sample, the increase in optical measurements could serve as noise from the plasma (i.e., the maximum possible noise). This increase was estimated from the intercept differences between 0% (i.e., standard) and 10% plasma concentration and can serve as the maximum level of noise derived from the plasma under this assumption. This increase was 0.128 (0.141–0.269) for 10% plasma, and thus, the value in normal plasma was 12.8 pg/ml. As plasma contains endogenous ghrelin, which increases optical absorption, we could only conclude that the maximal noise originating from the plasma must be smaller than this value. Thus, the nonspecific optical absorption from 10% plasma was relatively small for measuring the blood ghrelin level within the range observed in these monkeys. For instance, 12.8 pg/ml noise may come from 10% plasma within the entire range of 0–1,200 pg/ml in our measurements.

## Discussion

In this study, the consumption of food after 20 h of no food access elicited a decrease in blood unacylated ghrelin levels in three out of four monkeys. Blood ghrelin levels were further observed within various value ranges across individual monkeys, with most demonstrating a decrease in the value, whereas one monkey exhibited an increase in the value. This increase is surprisingly consistent in this monkey, indicating that the increase of blood ghrelin predicts food consumption in this particular monkey. In contrast, the blood osmolality level perfectly reflected the change in fluid balance in all monkeys after dry meal intake; the blood osmolality level consistently increased after dry meal intake for ∼3 h without fluid intake. Although blood osmolality and unacylated ghrelin levels can be used as indices for thirst and hunger, respectively, we need to consider the different characteristics of physical pressure and peptide hormone levels as hematological indices when evaluating the two satiety states.

### Measuring ghrelin as a physical index of hunger levels

In this study, we obtained evidence that ghrelin levels were high before food intake and then decreased after the food intake, reflecting hunger (Figs. 1*B*, 2*A*). This result is consistent with that of human studies demonstrating the role of ghrelin in food intake and its changes throughout the day (Spiegel et al., 2011). For instance, Spiegel et al. reported that an increase in unacylated ghrelin levels between meals in humans occasionally covaries with acylated ghrelin levels. Most human studies have reported average changes across participants (Spiegel et al., 2011; Marchio et al., 2019). In contrast, our repeated measurements from each monkey revealed individual changes in ghrelin levels and demonstrated the reliability of blood ghrelin levels as a physical index of hunger. However, it might be possible that the high ghrelin level before the first blood collection reflected an anticipatory response to food (Schussler et al., 2012).

Ghrelin plays a pivotal role in regulating food intake, body weight, and glucose metabolism in the brain (Tschop et al., 2000; Nakazato et al., 2001). Additionally, ghrelin is released from the stomach, where it transmits satiety signals to the central nervous system (Sato et al., 2014). Acylated and unacylated ghrelin in humans, monkeys, and rodents comprises 28 amino acid residues. In humans, acylated ghrelin is the active form that modifies the third serine with the fatty acid n-octane. The amino acid sequence of mammalian ghrelin is highly conserved in humans, monkeys, rats, mice, cows, pigs, and sheep. Furthermore, 10 amino acids from the N-terminus constitute the active region (Kaiya et al., 2011), which is used in ELISA to detect endogenous ghrelin in humans and monkeys. Unacylated ghrelin is detected in the stomach and blood at certain concentrations and does not bind to the growth hormone secretagogue receptor; however, some studies have reported that unacylated ghrelin enhances eating behavior (Toshinai et al., 2006).

Acylated and unacylated ghrelin (or total ghrelin) changed similarly in relation to consumption behavior in humans, whereas unacylated ghrelin was identified at a 10-fold higher concentration than acylated ghrelin (Spiegel et al., 2011). In addition, acylated ghrelin was discovered to be highly unstable in the whole blood and plasma. To acquire accurate ghrelin level data, blood samples were collected with ethylenediaminetetraacetic acid-aprotinin and centrifuged under cooled conditions within 30 min of collection (Hosoda and Kangawa, 2004). Following this protocol, we opted to measure unacylated ghrelin, which may serve as a physical index of hunger. We noted that acylated ghrelin, which is present at a concentration of ∼100 pg/ml in humans, was not measured in this study. We also noted that whether acylated ghrelin can be used as an index of hunger levels remains unclear.

### Blood osmolality measurement as a physical index of thirst levels

We discovered that the fluid balance of monkeys after eating a dry meal was reflected in their blood osmolality. In the water intake test, we confirmed that the monkeys consumed more water under the same conditions than that under the control condition, in which the monkeys ate no dry meal before water intake (Fig. 1*C*). The dry meal intake evoked the increase in the extracellular dehydration state, as measured by the osmolality level, indicated by how thirsty the monkeys were after eating the dry meal (Fig. 2*B*). This increase in osmolality (7 mOsmo/kgH_2_O) was almost equal to the osmolality increase observed under regular controlled water access in our previous study (8 mOsmo/kgH_2_O; Fig. 3; Yamada et al., 2010). In our previous study, we established that animals worked harder to earn more water on days when they exhibited lower hydration states (i.e., higher osmolality) than when they exhibited higher hydration states (i.e., lower osmolality). This relationship between osmolality level and water consumption behavior reflects the physiological condition of fluid balance, and it was observed as the increased osmolality and more water intake in the present study (Figs. 2*B*, 4*B*).

It is well known that osmoreceptors are located in the anterior–ventral third ventricle of the hypothalamus, where they detect changes in blood osmolality (Buggy et al., 1979). Neurons in the organum vasculosum of the lamina terminalis change their firing rates according to blood osmolality (Thrasher and Keil, 1987) to stimulate or suppress water and salt ingestion. In the present study, fluid balance changed after consuming a dry meal because the monkeys were not allowed access to water. We fed the monkeys certified diets containing water (8%), protein (21%), fat (7%), coarse ash (8%), crude fiber (3%), soluble nitrogen (53%), salt (0.7%), and vitamins. Monkeys consumed >130–160 g of this meal, which contained >1 g of salt. Salt intake without concurrent fluid intake must stimulate and change the body's fluid balance. Another important mechanism involves a reduction in blood volume, stimulates fluid ingestion, and suppresses diuresis via the renin-angiotensin system (Berne and Levy, 1993). Thus, osmolality is a critical component of the hydration state, and direct blood osmolality measurements provide a reliable index for quantifying the hydration state. We note that an increase in ghrelin levels may affect the drinking behavior of monkeys (Hashimoto et al., 2007).

### Age, sex, species, and experimental procedures

In our experiment, we used four macaque monkeys for blood tests, one of which exhibited distinct changes in blood ghrelin levels (Fig. 2*A*, Monkey SUN). We further analyzed the relationship between blood ghrelin and osmolality changes before and after dry meal intake (Fig. 3*A*), identifying no relationship, as these changes depended on the individual monkeys (Fig. 3*B*). Although ghrelin plays essential roles in adiposity and metabolism in vertebrates (Kaiya et al., 2013), the ghrelin levels were heterogeneous and differed between individuals. Multiple factors may be related to this discrepancy, and we discuss the potential factors that affect blood ghrelin levels. First, Monkey SUN, which exhibited increased blood ghrelin levels, differed from other monkeys in terms of age, species, and blood collection procedures. Monkey SUN was a rhesus macaque, whereas the others were Japanese macaques, although they are closely related species. This species difference is one possible explanation for this distinction. Sex differences cannot explain our results, as Monkey MIY was the only female among the four monkeys. As such, age is the most reasonable explanation for the difference: Monkey SUN was middle-aged (13 years old) compared with the other young monkeys (2–4 years old; see Materials and Methods). Indeed, ghrelin is a growth hormone, and total ghrelin and unacylated ghrelin levels decrease in an age-dependent manner (Wilasco et al., 2012). In aged frail adults, reduced levels of total ghrelin and impaired response to a meal test have been observed, suggesting that ghrelin contributes to the anorexia mechanisms associated with aging (Serra-Prat et al., 2009). Blood collection from Monkey SUN was performed while awake; however, age-related changes in ghrelin levels are one possible explanation of our results observed in Monkey SUN. In addition, a large number of individual observations across different ages are required to elucidate this issue. This inconsistency observed in one monkey (SUN) may limit the utility of ghrelin measures in evaluating hunger states.

### Neuroeconomic perspectives and satiety states

Hunger and thirst are distinct psychological (Powloski, 1953; Zimbardo and Montgomery, 1957; Ramachandran and Pearce, 1987) and physiological phenomena (Berne and Levy, 1993). However, they are interdependent (McKiernan et al., 2009), as observed in mammals and flies (Jourjine, 2017). From an economic perspective, this interdependence may explain why foods and beverages are complementary goods (Mas-Colell et al., 1995). From a food science perspective, foods and beverages contain similar components such as energy, water, salt, and other essential nutrients. The pathological association is often linked to obesity (Martens and Westerterp-Plantenga, 2012). From a neuroscientific perspective, interdependency is explained by the fact that the neural circuits underlying hunger and thirst interact with each other (B. J. Rolls, 1975; Jourjine, 2017; Eiselt et al., 2021; Tan et al., 2022). The interdependence was not observed in this study (Fig. 3), maybe because the interdependence might be observed when animals are both hungry and thirsty.

In neuroeconomic studies, hunger, thirst, and satiation have been demonstrated to have a significant impact on animal behavior (Minamimoto et al., 2012; Yamada et al., 2013; Yamada, 2017), although thirst and hunger may affect neural representations categorized as taste in the brain (Augustine et al., 2020; Pastor-Bernier et al., 2021). Physiologically, the hypothalamus plays an integral role in energy and water homeostasis by sensing and reacting to systemic calorie and osmotic fluctuations (Lee, 2016). Maintaining the energy and fluid balance is fundamental to survival; thus, a rebalance of these physiological states occur, such as dehydration after consumption of a dry meal in the present study.

For the reliable estimation of animal states, both indices should be measured with similar levels of precision. Herein, we aimed to compare the measurement precision and demonstrate the index reliability within their value ranges (Fig. 5) under the assumption. We still need to improve this evaluation, as well as the ELISA procedure. Additionally, easy access to blood samples is required to understand the relationship between neurophysiological measures and indices such as trial-by-trial correlations. However, blood ghrelin and osmolality measurements allow for an improved understanding of the neural basis of decision-making as a physical index of satiety. These measurements may be an easy and efficient way to evaluate controlled access to food and fluids in home cages.

## Data Availability

All data used in this study are presented in the manuscript.

## Synthesis

Reviewing Editor: Ifat Levy, Yale School of Medicine Decisions are customarily a result of the Reviewing Editor and the peer reviewers coming together and discussing their recommendations until a consensus is reached. When revisions are invited, a fact-based synthesis statement explaining their decision and outlining what is needed to prepare a revision will be listed below. The following reviewer(s) agreed to reveal their identity: Fabian Grabenhorst.

The authors have responded to all comments. The paper is improved and provides a methodological advance that will be of interest in the macaque neuroscience field. There are a few further points that should be addressed before publication.

1. Line 282-285: It is not clear why the authors state "this feeding interval of 20 h no-food yielded almost the same amount of dry meal's intake as that in regular feeding condition" but report significant results from two-way ANOVA test (two-way ANOVA, n = 104, d.f. = 92, duration, F = 13.6, P < 0.001; 285 monkey, F = 3.10, P = 0.032). Please provide further clarification.

2. Line 502-504: The authors discuss several factors that might explain individual differences in the results but it is unclear why age-related changes are considered the most likely factor to explain these observations.

3. Line 511: The authors mention the interdependence of thirst and hunger in the literature. However, in the main results, they report that osmolality changes and ghrelin changes are independent (Fig. 3). Additional discussion of this point would be helpful to try to reconcile these observations.

"However, they are interdependent (McKiernan et al., 2009), as observed in mammals and flies (Jourjine, 2017)."

4. References showing that hunger and satiety do not affect neuronal taste responses in gustatory cortex and nucleus of the solitary tract. These should be included to support the statement "Hunger and thirst may affect neural activity in specific brain regions,..."

Rolls, E. T., Scott, T. R., Sienkiewicz, Z. J., &Yaxley, S. (1988). The responsiveness of neurones in the frontal opercular gustatory cortex of the macaque monkey is independent of hunger. Journal of Physiology, 397, 1e12.

Yaxley, S., Rolls, E. T., Sienkiewicz, Z. J., &Scott, T. R. (1985). Satiety does not affect gustatory activity in the nucleus of the solitary tract of the alert monkey. Brain Research Brain Research Reviews, 347, 85e93.

## Author Response

Point-by-point reply to the reviewer's comments Manuscript No. eN-MNT-0481-23 "Method for evaluating hunger and thirst in monkeys by measuring blood ghrelin levels and osmolality" We greatly appreciate the reviewers' insightful feedback regarding our manuscript. The comments from the reviewer were critical and invaluable in helping us to improve the entire manuscript, especially our results which were greatly improved by the additional behavioral testing and analysis. We have carefully considered each comment and revised our manuscript accordingly, which has made our conclusions more compelling, clear, and strong.

In particular, we have obtained and analyzed eating and drinking behavioral data and have carefully addressed many of the points raised by the reviewer to improve the psychological description of hunger and thirst. We found the reviewers' comments to be both clear and reasonable, and hope that our revisions will facilitate an improved understanding of our goals and approach, particularly for behavioral evidence, which supports that our controlled food/water access is sufficient to lead to the hungry/thirsty state of monkeys. We are grateful for the help of the reviewer, and we appreciate that he/she can provide another evaluation of our revised manuscript.

Below, we have provided point-by-point responses to the reviewers' comments. As we have made several major and minor changes to our manuscript, we have included a list of all major structural changes in the figures below.

Figure 1: New Figure.

Figure 2: Old Figures 1 and 2 combined as Figure 2.

Figure 3: Old Figure 3 (Figure 3B in the current manuscript) with a new Figure 3A.

Figure 4: New Figure.

Figure 5: Old Figure 4.

2 Reviewer comments:

The study addresses the important question of how motivational states of hunger and thirst can be linked to physiological measures in monkeys in laboratory settings that are widely used in behavioral and neurophysiological experiments. The authors collected repeated blood samples from 4 monkeys before and after feeding, and measured plasma ghrelin and osmolality. The main finding is that plasma ghrelin levels decreased from pre- to post-feeding in 3 out of 4 monkeys while blood osmolality increased. The authors interpret these results in terms of changing hunger and thirst levels.

Implications for neuroeconomic studies are discussed.

The authors address an important topic - a new tool for measuring hunger and thirst will be very useful. However, the lack of behavioral data limits the interpretation of the physiological measures and the relevance of the findings for neuroscience. Moreover, the absence of behavioral data makes it difficult to rule out that Ghrelin levels before feeding reflected an anticipatory response to food rather than reflecting hunger per se. > We appreciate the reviewers' critical comments on our manuscript. We agree that the lack of behavioral results in the original manuscript limited our interpretation of the physiological measures of hunger and thirst; hence, their relationship to ghrelin and osmolality, respectively, was not obvious. In response to the reviewer's comments, we performed additional behavioral measures and their analyses to determine the level of thirst and hunger in the monkeys. We also conducted behavioral analyses to the data we have already obtained previously.

We have made all changes related to the critical points raised by the reviewer, as described below.

Major points:

1. The authors frequently refer to the 'hunger state' and 'thirst state' of the animals when they describe changes in the blood markers. However, no behavioral data that indicate thirst and hunger (e.g., increased or spontaneous initiation of water/food intake) are reported. Therefore, it is difficult to confirm whether the monkeys were adequately hungry after 20-hr fasting and indeed thirsty after dry meal intake. Without such behavioral confirmation, it is difficult to rule out that the increase in ghrelin does not just reflect an anticipatory response to the upcoming food, which has been reported in ghrelin (e.g., Schüssler et al., Obesity, 2012) and other metabolic hormones.

3 > We thank the reviewers for the valuable comment related to hunger and thirst levels as "whether the monkeys were adequately hungry after a 20-hr fast and indeed thirsty after consuming a dry meal?". This was really critical; thus, we have revised our manuscript to include extensive behavioral evidence indicating the monkeys' hunger and thirst. To achieve this aim, we made new behavioral tests, in addition to the analyses of the data we have already obtained. We explained these results below.

In this revision, we first confirmed that the monkeys were adequately hungry and thirsty in our experimental setting by performing additional behavioral measures in our test conditions, and then analyzed the amount of food intake for these tests.

We have added the behavioral measures to Figures 1 and 4. These figures confirmed the point raised by the reviewer "whether monkeys were hungry and thirsty in our experiment?". We elucidate these behavioral results below.

First, we confirmed that 20 h of no food access was sufficient to induce a hungry state in the monkeys. Here we assume that if monkeys ate regular amount of food after 20 h of no food access, monkeys would be hungry and the 20 h was enough to evoke hunger in each individual monkeys. As demonstrated in Figures 1B and C (the same as the figure below), we incorporated the amount of regular daily food intake, as a baseline for assessing and controlling their states. The four monkeys used for the blood test consumed 130-160 g of dry meal per day (Figure 1B, see Reg). In the test, the amount of food intake during the 1.5 h after the 20 h of no food access was almost equivalent to the amount of regular intake (Fig. 1B, see 20 h). In addition, 17 h of no food access may be sufficient to induce a heightened state of hunger in monkeys (see 17h).

For the thirst state, we measured the amount of water intake after eating a dry meal (Fig. 1C, see test) to confirm whether monkeys became thirstier after eating dry meals without water intake. These amounts were greater than those when the monkeys did not eat dry meals in the control condition (Fig. 1C; see con1).

Taken together, these behavioral results we added to the current manuscript suggest that the monkeys were hungry and thirsty under our controlled access to food and water. We have described this critical behavioral result by adding the following new sections:

Results, P11-12, Lines 270-292 4 "Effect of controlled food and water access on consumption behavior in monkeys To confirm that our experimental protocol obeying controlled access schedules adequately evoked hunger and thirst in monkeys, we prepared five different conditions in our experiment (Fig. 1A). First, in the blood test condition (Fig. 1A, second row), no food access was provided to the monkeys for 20 h. This controlled food access was started at 14:00 the day before the test in all test condition, except control 2 (Fig. 1A, bottom row).

This feeding interval of 20 h no-food yielded almost the same amount of dry meal's intake as that in regular feeding condition (Fig. 1B, see 20 h and Reg, two-way ANOVA, n = 104, d.f. = 92, duration, F = 13.6, P < 0.001; monkey, F = 3.10, P = 0.032), while the intake duration was 1.5 h for food consumption (Fig 1A, 2nd row, yellow bar). Indeed, 17 h of no food access we made as a pre-check (Fig 1A, bottom row, control 2) seemed to be sufficient to elicit this level of consumption behavior (Fig. 1B, see 17 h), although the effect of the no food duration differed between monkeys (interaction, F = 6.60, P < 0.001).

Thus, our controlled food access protocol used for the blood test (20h of no food access) adequately elicited a hunger state in the monkeys.

In the blood test, the monkeys consumed dry meals without any liquid reward (Fig.

1A, second row, gray bar for water). Under this feeding condition, monkeys became thirstier as indicated by the increased water intake (Fig. 1C, see test, see also Fig 1A, middle row) than those when they did not consume the dry meal (Fig. 1C, see con1, see also Fig 1A, fourth row, two-way ANOVA, n = 24, d.f. = 16, condition, F = 58.3, P < 0.001, monkey, F = 78.9) maybe because the dry meal contained almost no water but some sodium (See Materials and Methods). Conformation of this consumption behavior without anesthesia in the intake test indicated that under the blood test condition, which employs the same schedule as the intake test condition, monkeys were hungry and thirsty at the first and second blood collections, respectively." However, even though we confirmed the hunger and thirst states of the monkeys, we cannot rule out the possibility that changes in ghrelin levels reflect anticipatory responses to upcoming food delivery. Thus, we also mentioned the possibility of an anticipatory response to ghrelin in the Discussion because removing the anticipatory effect of food consumption in our test procedure was not possible.

P18, 420-421 "However, it might be possible that the high ghrelin level before the first blood collection reflected an anticipatory response to food after recovery (Schussler et al., 2012)." 5 We would again like to thank the reviewer for their comment.

2. Line 268: Similar to point 1, it is not sufficient to conclude that "the decrease in blood osmolality describes changes in the internal state of thirst" because (1) there was no behavioral quantification of thirst in this study and (2) ghrelin has also been shown to influence water intake, not just blood osmolality (Hashimoto et al., Endocrinology, 2007).

Thank you for your comments. As described in the first major comment, we measured the monkeys' drinking behavior using our testing procedure, and confirmed that monkeys became thirstier after eating the dry meals by the water intake increase (Fig. 1C same as the figure above).

Moreover, we also checked the correlation between the changes in ghrelin and osmolality (Fig. 3A, right figure), identifying no significant relationship between them. The result showed that changes in ghrelin and osmolality were not related with each other, indicating that ghrelin and osmolality did not reflect a single physical change in monkeys, rather different ones, such as hunger and thirst.

However, as the reviewer pointed out, we cannot rule out the possibility that changes in ghrelin may influence water intake, and thus, we discussed this possibility in the discussion as follows.

Discussion, P20, lines 471-472. "We note that an increase in ghrelin levels may affect the drinking behavior of monkeys (Hashimoto et al., 2007)" 3. The relevance to neuroscience fields is limited due to the lack of behavioral and neural measures. In particular, a clearer behavioral characterization of the effect of feeding and changing ghrelin levels is expected in a paper that situates these questions within the field of neuroeconomics and subjective values (Introduction, lines 101 etc.).

We appreciate the reviewers' precise evaluation of our work. Following the comments of the reviewer, we have now performed additional behavioral tests as well as additional analyses of behavioral and physiological data as described at 6 major comments No. 1 and 2, all of which support the ultimate goal of understanding economic issues. We believe that these changes will allow us to introduce the neuroeconomic issue in the Introduction section. If this is still not enough to relate the results to neuroeconomics, we hope to make the Introduction more toned down.

4. Ghrelin decreased in 3 of 4 monkeys but increased significantly in the 4th animal.

This inconsistency is not resolved in the current study (e.g., by behavioral differences in that animal). This would be important to resolve more conclusively, as the inconsistency potentially limits the use of the measure for behavioral and neurophysiological studies which rely on replicable effects in individual animals. > We appreciate the reviewer's insightful comment regarding the discrepancy between the monkeys. To examine this inconsistency further, we performed an additional analysis (Fig. 3A, right same as above), which described the relationship between changes in ghrelin and osmolality. This analysis, as suggested by the reviewer in comment no. 7, must determine individual differences and overall relation in ghrelin and osmolality in monkeys. Overall, no relationship was observed between changes in ghrelin osmolality before and after the dry meal intake. If we look at individual data (please compare the distribution of different colors), the data points from all four monkeys, demonstrated almost no relationship in each individual.

We also analyzed the behavioral change by examining whether food and water intake behavior explained individual differences, but we did not identify any clear evidence to support this idea (Figure 4C, see right). Thus, we did not find positive results to answer the reviewer's query. After obtaining these two new results, we added the following description to the Results and Discussion section:

Results, P14, Lines 331-334 "We further examined the relationship between changes in blood osmolality and ghrelin levels to determine whether these values were related, identifying no relation between these changes (Fig. 3A; linear regression, n = 32, df = 30; intercept, t = 9.54, P < 0.001; ghrelin difference, t = 1.30, P = 0.20)." Results, P14-15, Lines 341-344 7 "We further examined whether a behavioral relationship was present between food intake and an increase in water intake under non-anesthetized conditions (Figs. 4A and B), but did not detect any such relationship (Fig. 4C)." Discussion, P20, lines 476-478 "We further analyzed the relationship between blood ghrelin and osmolality changes before and after dry meal intake (Fig. 3A), identifying no relationship, as these changes depended on the individual monkeys (Fig. 3B)." As we did not detect any clear reason to explain the individual differences, we have also added a discussion on this issue that weakened our conclusion.

Discussion, P21, Lines 495-497 "This inconsistency observed in one monkey (SUN) may limit the utility of ghrelin measures in evaluating hunger states." 5. Related to the above points, the conclusion from the abstract that "ghrelin and osmolality may provide a precise reflection of the physiological states of individual monkeys for hunger and thirst" is insufficiently supported by data due to the lack of behavioral measures to confirm motivational states of hunger and thirst; the claim of "a precise reflection" in particular does not seem to be well supported. > We appreciate the reviewer's suggestions. Despite incorporating additional behavioral data, we have opted to soften this point by removing the term "precise," as follows.

Abstract, P3, lines 66-68 "Thus, ghrelin and osmolality may reflect the physiological states of individual monkeys regarding hunger and thirst, suggesting that these indices can be used as tools for monitoring hunger and thirst levels that mediate an animal's decision to consume rewards." 6. Line 147: Please provide detailed ingredients of the dry meal, including the calorie and sodium content, because blood osmolality and thirst level together influence the 8 motivation for consuming sodium-containing food and liquids.

Thank you for your comments. This is an important aspect of our experimental design. We added this information in the Method section as follows.

Methods, P7, lines 148-152 ‘In the approved controlled food access protocol, the monkeys received a fixed daily allocation of dry meals (certified diet, PS-A, Oriental, Japan), containing water (8.0 {plus minus} 0.7 g), protein (21.5 {plus minus} 0.3 g), fat (6.8 {plus minus} 0.3 g), ash (7.7 {plus minus} 0.2), fabric (3.1 {plus minus} 0.3), soluble nitrogen’free (52.9 {plus minus} 0.7), calorie (359.1 {plus minus} 3.5 kcal) per 100 g. The diet also contains sodium (0.66 g per 100 g), vitamins, and other minerals." 7. The relationship between changes in blood ghrelin and blood osmolality in Figure 3 is difficult to interpret, and statistical tests are required to conclude tha changes in blood ghrelin and blood osmolality are truly independent, as stated in line 280.

Alternatively, correlations between 'changes of values' might be more meaningful. For example, one could plot regression lines between 'ghrelin changes' and 'osmolality changes' before and after meals, either pooled across monkeys or separately for each monkey. Please also report the statistical significance of the regression and the slope differences across monkeys. > As the reviewer suggested, we have added the regression analysis in the new Figure 3A (see right, same figure as shown above), which showed no relation between them. We have also retained the original figure to explain the differences between the monkeys in Figure 3B. We have also added a statistical description to explain this figure in the Results section as follows:

Result, P14, Lines 331-334 "We further examined the relationship between changes in blood osmolality and ghrelin levels to determine whether these values were related, identifying no relation between these changes (Fig. 3A; linear regression, n = 32, df = 30; intercept, t = 9.54, P < 0.001; ghrelin difference, t = 1.30, P = 0.20)." These additions have significantly enhanced the clarity of our results. Thank you again for your comment.

9 8. Line 106: "Because hunger and thirst may dramatically change neural activity in most parts of the brain (de Araujo et al., 2006)". This statement is contradicted by evidence from monkeys that motivational status does not affect single-neuron activity in NTS, insula, frontal operculum (works by Rolls, Pritchard, Scott). > We thank the reviewer for their suggestion. As the reviewer suggested we tried to add the reference and change the introduction as follows.

Introduction, P5, lines 106-107 "Hunger and thirst may affect neural activity in specific brain regions, or dramatically change the activity in many parts of the brain (de Araujo et al., 2006). " As we did not find the reference suggested by the reviewer, we are happy if they could provide us with more direct information.

9. Line 108: "The subjective values of the items used in the monkey experiments depend on these physiological statuses; for example, the intake of juice rewards elicits changes in both statuses" No reference is provided to support these statements. > We would like to thank the reviewers for their comments regarding our descriptions. As we made this speculation based on our experiences, and we lack a suitable reference for this description, we have amended our statement as follows:

Introduction, P5, lines 109-111 "The subjective values of the items used in the monkey experiments depend on these physiological statuses. For instance, the intake of juice rewards must elicit changes in both statuses, since it contains both sugar and liquid" 10. Line 267: "Thus, the blood osmolality level was capable of consistently describing changes in the internal state of thirst in all four animals" No evidence is provided that blood osmolality is related to thirst, which is a motivational state defined in terms of 10 behavior. > To address the reviewer's point, we have added the following description of behavior, as we obtained behavioral evidence from the additional experiment: monkeys drink more water if they ate dry meals (Fig. 1C, test) compared to that observed with no food consumption (con 1). This indicates that dry meal intake evoked thirst.

Result, P14, lines 327-330 "Thus, the blood osmolality level was capable of consistently increasing the internal state of thirst in all four animals (Figs. 1C and 2 B), which is consistent with the results of our previous studies (Yamada et al., 2010; Minamimoto et al., 2012; Yamada et al., 2013)." 11. The authors neglect parts of the relevant literature, e.g. in line 423 relevant papers by Pastor-Bernier and Schultz, 2021, PNAS "Reward-specific satiety affects subjective value..." and by Rolls et al. are not discussed. > We refer to the following paper in the Introduction section:

Introduction, P4-5, lines 94-97 "These physical indices of hunger and thirst are useful for assessing and controlling the motivational state of primates, in addition to other possible measures, such as accumulated earned rewards (Minamimoto et al., 2012; Pastor-Bernier et al., 2021)." We also added the reference to discussion as follows:

Discussion, P21, lines 512-513 "although thirst and hunger may affect neural representations categorized as taste in the brain (Augustine et al., 2020; Pastor-Bernier et al., 2021)" Minor points:

12. Line 246: Please discuss and justify why you measured unacylated ghrelin instead 11 of the biologically active acylated ghrelin or total ghrelin.

The reviewer raised a question regarding why we measured unacylated ghrelin instead of acylated or total ghrelin. We opted for this choice due to several reasons.

One reason for this is that the only ELISA kit available for monkeys assessed unacylated ghrelin. Another reason is that acylated ghrelin is known to be present in very small amounts (10-fold differences) compared to the amount of unacylated ghrelin in humans; thus, we decided to measure unacylated ghrelin. To explain the reason for measuring unacylated ghrelin, we have added the following description to the Discussion section, as the reviewer suggested:

Discussion, P18-19, lines 434-443 "Acylated and unacylated ghrelin (or total ghrelin) changed similarly in relation to consumption behavior in humans, whereas unacylated ghrelin was identified at a 10-fold higher concentration than acylated ghrelin (Spiegel et al., 2011). In addition, acylated ghrelin was discovered to be highly unstable in the whole blood and plasma. To acquire accurate ghrelin level data, blood samples were collected with ethylenediaminetetraacetic acid-aprotinin and centrifuged under cooled conditions within 30 min of collection (Hosoda and Kangawa, 2004). Following this protocol, we opted to measure unacylated ghrelin, which may serve as a physical index of hunger. We noted that acylated ghrelin, which is present at a concentration of approximately 100 pg/mL in humans, was not measured in this study. We also noted that whether acylated ghrelin can be used as an index of hunger levels remains unclear." 13. Line 291: Please provide reference or report in detail your previous evaluation of measurement error for osmolality (2 mOsm/kgH2O). Also, please make clear that "the measurement error" in line 291 refers to the error for osmolality, different to the next sentence about ghrelin.

We thank the reviewer for this comment. In this error evaluation using our method, we assessed three samples from a single collected sample, while 2 mOsm/kgH2O was the maximum difference observed in our previous study (Yamada et al., 2010).

This is a robust observation. We have added this description to the Methods section as follows:

12 Methods, P9, Lines 210-212 "This error was determined by measuring three samples from a single collected sample, with 2 mOsm/kgH2O being the maximum difference observed in our previous study (Yamada et al., 2010)." As the reviewer pointed out, we also changed the method section as follows.

Methods, P15, lines 355-357 "For the osmolality measures we employed, we previously evaluated the measurement error of osmolality, as the value range from a single sample was within almost 2 mOsm/kgH2O in our procedure.." 14. Line 293: Please provide reference for the statement that "plasma has a nonspecific effect on optical absorption".

Thank you for this comment. We added the following references:

Results, P15, Lines 358-360 "As plasma must induce a nonspecific effect on optical absorption, such as the nonspecific binding of proteins (Neutens et al., 2021), we evaluated this noise as follows:" 15. Line 407: It is not clear why you come to the conclusion that age is the most likely explanation for differences across animals rather than different experimental conditions (e.g. blood sampling techniques). Please discuss more the potential influence of different procedures on the measurement levels (e.g., duration between blood sampling to measurement due to the instability of ghrelin).

We thank the reviewer for this comment. Indeed, we cannot rule out these alternative possibilities, and we have therefore added a discussion regarding the instability of ghrelin and tempered our conclusion as follows:

Discussion, P18, Lines 436-439 "In addition, acylated ghrelin was discovered to be highly unstable in the whole blood and plasma. To acquire accurate ghrelin level data, blood samples were collected with ethylenediaminetetraacetic acid-aprotinin and centrifuged under cooled conditions within 13 30 min of collection (Hosoda and Kangawa, 2004)." 16. Line 433: In the discussion, you point out the need to measure physiological states in monkey neurophysiological studies, particularly of the reward system. Please also discuss how you think this method is feasible or "easy", as stated in line 436, for daily monkey neurophysiological experiments, which may require repeated measurements of hunger and thirst levels to correlated with trial-by-trial behavior and neuronal activities.

We thank the reviewer for this comment. We have added the following discussion to the manuscript:

Discussion, P22, Lines 520-523 "We still need to improve this evaluation, as well as the ELISA procedure. Additionally, easy access to blood samples is required to understand the relationship between neurophysiological measures and indices such as trial-by-trial correlations." 17. Please include statistical significance levels in the legends of figures 1-3. > As it is not easy to represent results of the ANOVA on these figures and their legends, we did not include these significance levels in the figures and their legends. Instead, we have included precise statistical descriptions of the text in the results corresponding to each figure.

18. Please standardize the monkey names in the main texts and figures ('MI') and in the methods section ('MIY') to avoid confusion. > We have corrected this to MIY.

19. Line 81: "cravings" may not be the accurate term here. > We have revised the cravings as follows:

14 Introduction, P4, lines 80-81 "Hunger and thirst levels are subjectively evaluated and controlled by experimenters, yet these states vary individually due to differences in homeostatic conditions." 20. Line 88: "control of either one mostly affects the other status" please consider rephrasing for clarity. > We have rephrased the sentence as follows:

Introduction, P5, lines 97-100 "However, the lack of a simultaneous assessment of hydration and metabolic status leads to difficulty in evaluating neural correlates for hunger and thirst in behaving monkeys, as control of either hunger or thirst can affect the status of the other (Ramachandran and Pearce, 1987; Watts, 1999; Betley et al., 2015)." 21. Line 233: consider rephrasing 'blood ghrelin levels and blood osmolality' for clarity. > We have corrected it. Additionally, we also examined whether a similar description exists.

22. Line 134: "corrected" should be "collected" > This has been corrected.

23. Line 138: consider adding spaces between "ethylene diamine tetra-acetic acid" > We have added spaces. Thank you for your comment.

24. Line 226: "intercept valuess" should be "intercept values" 15 > We have removed the "s".

25. Line 427: "Our results observed in the body fluid balance indicated that maintaining energy and water balance is fundamental to survival". I don't see how this can be concluded from the current data. > We have removed this description and revised it as follows:

Discussion, P21-22, lines 515-517 "Maintaining the energy and water balance is fundamental to survival; thus, a rebalance of these physiological states occur, such as dehydration after consumption of a dry meal in the present study." 26. Line 437: "homepage" should be "homecage" > This has been corrected.
